# Cost-effectiveness analysis of two inhaled antibiotics for stable Bronchiectasis with Pseudomonas aeruginosa infections in China

**DOI:** 10.1371/journal.pone.0324254

**Published:** 2025-06-18

**Authors:** Wanxin Chen, Xuerong Chen, Liting Lai, Bin Hong, Canghong Zhi, Honglin Li, Sha Li, Jie Jiang

**Affiliations:** 1 College of Pharmacy, Jinan University, Guangzhou, China; 2 Department of Respiratory Medicine, The Third People’s Hospital of Shenzhen City, Shenzhen, China; 3 Department of Respiratory and Critical Care Medicine, Zhangzhou Second Hospital, Zhangzhou, China; 4 Joincare Pharmaceutical Group Industry Co., Ltd., Shenzhen, China; Sutter Gould Medical Foundation, UNITED STATES OF AMERICA

## Abstract

**Objective:**

To evaluate the cost-effectiveness of two available options for inhaled antibiotic treatment for patients with Bronchiectasis (BE) with *Pseudomonas aeruginosa* (PA) infections from the perspective of China’s healthcare system.

**Methods:**

A four-state Markov model was developed over a one-year horizon to simulate the cost-effectiveness of two inhaled antibiotic strategies: Tobramycin inhalation solution (TIS) versus nebulized colistimethate sodium (CMS). The inputs for the model were derived from phase III clinical trials and published literature, with cost data were sourced from public and real-world databases, etc. The incremental cost-effectiveness ratio (ICER) was assessed, setting the willingness-to-pay threshold at one times the per capita GDP of China. Scenario and sensitivity analyses were performed to explore the impact of uncertainties in input parameters.

**Results:**

Over a one-year period, TIS was found to dominate CMS, resulting in a cost saving of CNY 41,109.53 (USD 5,689.27) and an increase of 0.0048 quality-adjusted life years (QALYs) per patient. Sensitivity analyses confirmed the robustness of these findings, which remained consistent under various scenarios.

**Conclusions:**

TIS reduces healthcare costs and improves clinical outcomes compared to CMS in managing BE with PA infections in China. This study supports the inclusion of TIS in clinical guidelines for managing BE with PA infections, considering both economic benefits and health outcomes.

## Introduction

Bronchiectasis (BE) is a chronic respiratory condition characterized by irreversible dilation of the bronchi and sustained bacterial infections. A comprehensive analysis of data from China’s Urban Employee Basic Medical Insurance and Urban Resident Basic Medical Insurance, covering over 380 million individuals aged 18 and above, revealed that the prevalence of BE among Chinese adults escalated from 75.48 per 100,000 in 2013 to 174.45 per 100,000 in 2017, marking a 131% increase [1]. Simultaneously, the annual hospitalization cost per patient rose from $3,023.05 in 2013 to $3,470.86 in 2017, thus highlighting a significant economic burden on the healthcare system. *Pseudomonas aeruginosa* (PA), isolated in 9% to 33% of BE patients [2], emerges as the primary infectious agent during acute exacerbations [3,4]. Chronic colonization by PA significantly escalates the risk of acute exacerbations, hospitalization, and mortality [5,6].

Research findings suggest that eradicating PA could potentially improve patient outcomes [7,8], and inhaled antibiotics are effective in eradicating PA [9], reducing the risk of acute exacerbations, and hospitalization [5]. Recognizing the severity of these infections, esteemed health organizations such as the British Thoracic Society [6], the European Respiratory Journal [8], and the Infectious Diseases Group of the Respiratory Diseases Branch of the Chinese Medical Association have issued guidelines advocating for pathogen eradication in adults with chronic PA infections associated with BE. Recommended treatment regimens emphasize oral or intravenous antibiotics alongside inhaled antimicrobial agents, specifically highlighting inhaled antibiotics as preferred options due to its specific drug targets and minimal systemic adverse effects. In China, the development of inhaled antibiotics is still in its nascent stage. Currently, the available options for inhaled antibiotic treatment for patients with BE accompanied by PA infection are limited to Tobramycin inhalation solution (TIS) and colistimethate sodium (CMS) [10].

A pivotal 16-week multicenter, randomized, double-blind, placebo-controlled Phase III clinical trial conducted in China (NCT03715322) demonstrated that TIS significantly enhances outcomes in patients with stable BE accompanied by PA infections. Notably, the trial reported changes in PA sputum density (adjusted mean difference of 1.74 log_10_ colony-forming units/g; 95% CI, 1.12–2.35, *P* < 0.001) and Quality-of-Life Bronchiectasis Respiratory Symptoms scores (adjusted mean difference, 7.91; 95% CI, 5.72–10.11, *P* < 0.001). The clearance rate of PA was notably higher in the tobramycin group compared to the placebo group (29.3% vs 10.6%) [11].

Nebulized CMS is other available options for patients with stable BE accompanied by PA infections, and it has been used in many clinical cases in China [12,13]. It is recommended as an alternative medication for first-line therapy in China [14]. Therefore, we aimed to evaluate the cost-effectiveness of TIS compared with nebulized CMS as a prolonged inhalation therapy for patients with stable BE accompanied by PA infections in China.

## Materials and methods

### Model design

A four-state Markov model was developed using Microsoft Excel to assess the cost-effectiveness of TIS compared to nebulized CMS for treating adults with stable BE with PA infections, from the perspective of China’s healthcare system.

The cycle length of the model was set at four weeks, incorporating a half-cycle correction to improve accuracy. Given that extended study periods can introduce uncertainty and potential deviations from actual outcomes, a one-year time horizon was deemed most appropriate for this analysis. Cost-effectiveness is measured by the incremental cost-effectiveness ratio (ICER), which quantifies the cost per additional quality-adjusted life year (QALY) gained with TIS relative to CMS.

In alignment with the China Guidelines for Pharmacoeconomic Evaluations [15], the calculated ICERs were compared against a willingness-to-pay (WTP) threshold of CNY 89,358.00 (USD 12,366.52) per QALY, corresponding to China’s per capita gross domestic product (GDP). Annual discount rates were set at 5% for both costs and health outcomes, with currency conversions based on the June 2023 exchange rate of 1 USD = 7.2258 CNY.

### Model structure

**Fig 1:**
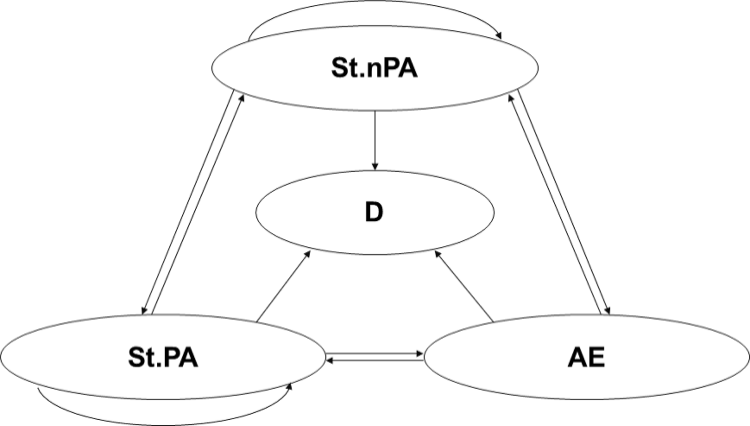
Markov model representation. Stable with PA (St.PA): refers to stable patients with positive sputum cultures for PA. This is the starting state of all cohorts. In the next cycle, some surviving patients may transition to stable or without PA infection, acute exacerbation, or remain in their current state. Stable without PA (St.nPA): refers to stable patients with negative sputum cultures for PA. In the next cycle, some surviving patients may transition to either stable with PA infection, acute exacerbation, or remain in their current state. Acute Exacerbation (AE): refers to patients in the acute exacerbation phase. The survivors will return to a stable state in the next cycle. Death (D): refers to deceased patients. This is an absorbing state.

The model structure was built according to the disease process of bronchiectasis, which was also verified by clinical experts. Four health states were defined with an initial state of stable-stage without PA infections: stable without PA infections, stable with PA infections, acute exacerbation, and death. Fig 1 illustrates the transition relationships between these states. Based on clinical expert opinion [14], the duration of an acute exacerbation typically does not exceed two weeks; therefore, it is assumed that all patients in this state return to a stable condition after one cycle.

### Model inputs

#### Target population.

The target population comprises Chinese adults diagnosed with BE associated with PA infections. This model reflects patient characteristics consistent with those observed in the Phase III clinical trial of TIS, specifically an average age of 53 years and 44% being male [11].

#### Treatment options.

The medication plans for the intervention group and the control group were based on the dosages mentioned in the literature [11,16]. Both patient groups underwent two courses of inhalation therapy, each consisting of 28 days on-treatment followed by 28 days off-treatment, totaling 56 days of medication annually. This aligned with the treatment durations recommended by Chinese expert consensus. The model included prolonged inhalation treatment with either TIS or CMS, prescribed as follows:

- TIS: 300 mg twice daily.
- CMS: 1 million units twice daily.

Additionally, during both on-treatment and off-treatment phases, patients in stable conditions consistently received an expectorant regimen, specifically *N*-acetylcysteine tablets (600 mg, twice daily).

#### Clinical data.

A Phase III clinical study involving 357 stable BE patients with PA infections used a mesh nebulizer to administer either TIS (300 mg/5 ml, twice daily, *N* = 167) or saline solution (5 ml, twice daily, *N* = 172). The treatment of nebulized TIS, conducted over two cycles (each including 28 days on and 28 days off treatment), significantly decreased PA density and notably enhanced patient quality of life, with a higher rate of PA sputum culture negativity (Day 29: 29.3% *vs.* 10.6%, *P* < 0.01) [11]. Adverse event rates were comparable between the groups. Based on the data collected in this clinical trial, the dynamics of PA clearance and transition probabilities between “with PA” and “without PA” states were calculated every four weeks for surviving patients, as detailed in S1 Table. Additionally, 16% (36/167) of the patients treated with TIS experienced acute exacerbations within 16 weeks post-medication, marking an exacerbation rate of 22%.

Since there is no head-to-head randomized controlled trial comparing TIS with nebulized CMS for the treatment of Chinese patients with BE with PA infections, the efficacy of nebulized CMS in Chinese patients was estimated based on the efficacy of TIS and the odds ratio (OR) between the two drugs. A RCT evaluated the effectiveness of inhaled tobramycin and CMS in eradicating PA in patients with non-cystic fibrosis bronchiectasis, showing an OR of 1.40 (95% CI: 0.36, 5.35) [16,17].

#### Transition probability.

Transition probabilities between disease states in the model were sourced from clinical trials, cohort studies, and data provided by the Chinese Bureau of Statistics. The transition probabilities for each 4-week cycle were calculated using the formula *tp* = 1 – exp(-*rt*), detailed in Table 1.

**Table 1:** Parameters of transition probabilities per cycle.

Parameters	Base-case value (Range)	Distribution	Note/source
TP between PA infections and non-PA infections of TIS used in patients within stable state			
Pa2nonPa.4w	29.30% (26.37%, 32.23%)	Beta (70, 170)	Statistical data from Phase III clinical trials of TIS (NCT03715322 [11])
Pa2nonPa.8w	4.35% (3.91%, 4.78%)	Beta (96, 2103)	
Pa2nonPa.12w	17.27% (15.55%, 19.00%)	Beta (83, 395)	
Pa2nonPa.16w	7.29% (6.56%, 8.02%)	Beta (93, 1178)	
nonPa2Pa.8w	62.86% (56.57%, 69.14%)	Beta (37, 22)	
nonPa2Pa.12w	29.41% (26.47%, 32.35%)	Beta (70, 169)	
nonPa2Pa.16w	35.48% (31.94%, 39.03%)	Beta (64, 117)	
OR for PA clearance: CMS versus TIS	1.40 (0.36, 5.35)	Log Normal (0.34, 0.69)	[17]
TP of Pa2AE	5.89% (5.30%, 6.48%)	Beta (94, 1503)	[11]
HR for Acute Exacerbation: PA Infections versus Non-PA Infections	2.40 (1.20, 4.79)	Log Normal (0.88,0.35)	[18]
HR for Mortality: PA Infections versus Non-PA Infections	3.07 (1.32, 7.15)	Log Normal (1.12,0.43)	[18]
HR for Mortality: Severe versus Mild Cases	14.84 (1.93, 114.24)	Log Normal (2.70,1.04)	[19]

TP, transition probability. PA, *Pseudomonas aeruginosa*. Pa, PA infections. 2, to. nonPa, non-PA infections. TIS, tobramycin inhalation solution. CMS, colistimethate sodium. OR, Odd ratio. HR, Hazard ratio.

For BE patients, PA infections were independently associated with an increased risk of acute exacerbations and mortality. A retrospective study involving 1,188 Chinese patients with stable BE indicated a significant relationship between PA infections and higher rates of acute exacerbations, with an odds ratio (OR) of 2.40 (95% CI: 1.20 to 4.79). Additionally, PA infections were found to substantially contribute to all-cause mortality, with a hazard ratio (HR) of 3.07 (95% CI: 1.32 to 7.15) [18]. It was assumed that the mortality rate for patients with stable BE without PA infections aligned with the general mortality rates in China, as reported in the “China Statistical Yearbook 2021” [20]. Acute exacerbations were also noted as a significant mortality risk factor. A prospective cohort study in Singapore, primarily involving outpatient BE patients (over 80% Chinese), demonstrated that severe BE significantly increases mortality risk, with a risk ratio of 14.84 (95% CI: 1.929 to 114.235) [19]. For more details, refer to Table 1.

#### Adverse events.

No studies have demonstrated the comparative safety of TIS and nebulized CMS in Chinese patients with bronchiectasis. A Phase III RCT investigating TIS for stable bronchiectasis patients in China revealed no significant difference in treatment-related adverse events compared to placebo, indicating that the use of this medication does not lead to an increased occurrence of additional adverse events [11]. Furthermore, an observational study conducted on Caucasian individuals receiving treatment also reported no significant disparity in treatment-related adverse events between nebulized tobramycin and nebulized CMS [21]. Thus, this analysis did not consider the impact of adverse events on cost and utility values.

#### Utility data.

It was revealed that the EQ-5D-5L utility value for Chinese BE patients within acute exacerbation was 0.55, and for patients with PA infections transitioning to the stable state, the EQ-5D-5L utility value was 0.77 [13]. The EQ-5D-5L utility value of Korean patients (which were also Asian) with BE in a stable state was reported to be 0.87, according to another database study [22]. A summary of these utility values for each health state is provided in Table 2.

**Table 2:** Parameters of health utility values for each state.

Parameter	Base-case value (Range)	Distribution	Source
Utility: Stable state without PA	0.87 (0.78, 0.96)	Beta (12, 2)	[22]
Utility: Stable state with PA	0.77 (0.69, 0.85)	Beta (22, 7)	[13]
Utility: Acute exacerbation	0.55 (0.50, 0.61)	Beta (44, 36)	[13]
Utility:Death	0	/	/

PA, *Pseudomonas aeruginosa*. D, Death

#### Cost data.

Only direct medical costs were included in this study due to the perspective of the Chinese healthcare system. Based on the assumption mentioned in the part of *Treatment Options*, the direct medical costs included: costs of drug acquisition and administration, costs of examination and laboratory test, outpatient visit costs, and hospitalization costs. And all costs sourced from literature were converted to 2023 (CNY, ¥) using the Chinese Consumer Price Index (CPI) for health care.

Drug acquisition costs were derived from the listed prices in the Yaozh database, and the costs of medical services were calculated based on the median prices across seven cities in China. During the acute exacerbation phase, costs were sourced from post-marketing clinical studies [13] and research on the disease burden associated with BE [23].

Most patients (95%) incurring an acute exacerbation would initially visit the outpatient clinic directly, incurring an average treatment cost of CNY 1,263.65 (USD 174.88) [23] per patient. If outpatient treatment failed to control symptoms effectively, approximately 47% of these patients would subsequently require hospitalization [24], which resulted in average inpatient costs of CNY 26,017.86 (USD 3,600.69) [1].

All patients received two course of prolonged inhalation treatment in the stable phase per year throughout the study duration. Detailed calculations of these drug treatment costs, including the routine use of expectorants, are outlined in S2 Table. For patients in stable states, the outpatient-visit costs included consultation fees and routine examination and testing fees, essential for disease management. These routine examinations and tests encompassed complete blood counts, C-reactive protein tests, sputum cultures, antibiotic susceptibility tests, and lung function tests.

It is assumed that all patients in stable states within prolonged inhalation treatment visited the clinic every two weeks for a new 14-day prescription, and they underwent routine examinations and tests after each 4-week treatment. During the periods of medication withdrawal, patients with stable BE rarely visited the clinic, with the annual number of outpatient visits for these patients with stable BE estimated at approximately 0.9 times per year, as reported in the literature [1].

All parameters used for calculating direct medical costs are comprehensively presented in Table 3.

**Table 3:** Parameters of costs.

Parameter	Base-case value (Range)	Distribution	Source/Note
Medication cost of Baseline Treatment, per cycle	¥259.28 (233.35, 285.21)	Gamma (100, 3)	The median prices listed in the Yaozh database
Outpatient consultation fee of Baseline Treatment, per cycle	¥1.73 (1.55, 1.90)	Gamma (100, 0)	Median prices of medical services in 7 cities; [1]
Outpatient routine examination fee of Baseline Treatment, per cycle	¥12.14 (10.93, 13.35)	Gamma (100, 0)	
Drug acquisition and management cost of TIS, per cycle	¥14,201.60 (14,201.60, 14,201.60)	Constant (0,0)	Healthcare reimbursement prices
Drug acquisition and management cost of CMS, per cycle	¥35,311.01 (21,498.40, 38,314.08)	Constant (0,0)	
Outpatient consultation fee of inhaled treatment-on, per cycle	¥50.00 (45.00, 55.00)	Gamma (100, 1)	Median prices of medical services in 7 cities
Outpatient routine examination fee of inhaled treatment-on, per cycle	¥175.83 (158.24, 193.41)	Gamma (100, 2)	
Outpatient treatment rate for patients with acute exacerbations	95.00% (85.50%, 100.00%)	Beta (4, 0)	[25]
Per capita outpatient treatment cost for acute exacerbations	¥1,263.65 (1,137.29, 1,390.02)	Gamma (100, 13)	[23]
Admission rate for patients with acute exacerbations	46.99% (42.29%, 51.69%)	Beta (53, 59)	[24]
Per capita inpatient treatment cost for acute exacerbations	¥26,017.86 (23,416.07, 28,619.65)	Gamma (100, 260)	[1]

TIS, tobramycin inhalation solution. CMS, colistimethate sodium

### Uncertainty analysis

#### One-way deterministic sensitivity analysis.

One-way deterministic sensitivity analysis was conducted to ascertain the model’s robustness by variably adjusting the parameters, either by modifying the 95% confidence intervals or by applying a 10% increase or decrease to the input values. This approach helped identify which parameters the model is most sensitive to. Additionally, the discount rate was set within a range, from a minimum of 0% to a maximum of 8%. The net monetary benefit (NMB) was calculated as part of this analysis, defined as the incremental QALYs multiplied by the WTP threshold per QALY, minus the incremental costs. The results of this univariate sensitivity analysis are depicted in a tornado diagram.

#### Probabilistic sensitivity analysis.

Probabilistic Sensitivity Analysis (PSA) was performed to assess the uncertainty across the entire model comprehensively. This analysis involved taking 5,000 random samples from the designated parameter distributions (refer to Tables 1–3) using Monte Carlo simulation techniques. The outcomes were displayed in the ICER scatter plot and a cost-effectiveness acceptability curve (CEAC), providing a visual representation of the findings.

#### Scenario analysis.

Scenario analyses were carried out to evaluate the impact of varying durations of time horizon on the result. This scenario subjected patients to a long-term simulation with a time horizon set from 5 to 20 years. All other settings from the base analysis were maintained. Drawing on data from Phase III clinical trials, the average age of BE patients was 53 years [11]. Literature on the five-year mortality rate for BE patients [26] suggested that the typical death age was around 73 years, indicating that a 20-year maximum time horizon was sufficient to encompass the lifespan of most patients within the study.

### Ethics statement

This article is based on previously conducted studies and does not contain any new studies with human participants or animals performed by any of the authors. All analyses were derived from publicly available data and existing literature, ensuring compliance with ethical standards in the absence of direct human or animal experimentation.

## Results

### Base-case analysis

Over the course of a 1-year simulation, TIS was found to dominate nebulized CMS, yielding an additional 0.0048 QALYs while saving CNY 41,109.53 (USD 5,689.27). The total costs amounted to CNY 38,549.50 (USD 5,334.98) for TIS compared to CNY 79,659.03 (USD 11,024.25) for nebulized CMS. TIS was demonstrated cost reductions in anti-PA medication, as well as outpatient and hospitalization expenses during periods of acute exacerbation. At the WTP threshold of CNY 89,358.00 (USD 12,366.52) per QALY, the net monetary benefit for TIS was calculated to be CNY 41,542.26 (USD 5,749.16), as detailed in Table 4.

**Table 4:** Base-case analysis results.

Items	TIS	CMS	Increments
Total costs	¥38,549.50	¥79,659.03	¥-41,109.53
Inhaled antibiotics medication cost	¥27,538.77	¥68,424.59	¥-40,885.82
Stable state baseline treatment cost	¥3,510.05	¥3,504.68	¥5.37
Acute exacerbation outpatient cost	¥670.68	¥691.16	¥-20.48
Acute exacerbation hospitalization cost	¥6,830.00	¥7,038.60	¥-208.60
QALYs	0.75	0.75	0.0048
Costs Increments	¥-41,109.53		
QALYs Increments	0.0048		
ICER (Yuan/QALY)	TIS dominant.		

QALYs, quality-adjusted life years. ICER, incremental cost- effectiveness ratio. TIS, tobramycin inhalation solution. CMS, colistimethate sodium

### One-way sensitivity analysis

**Fig 2:**
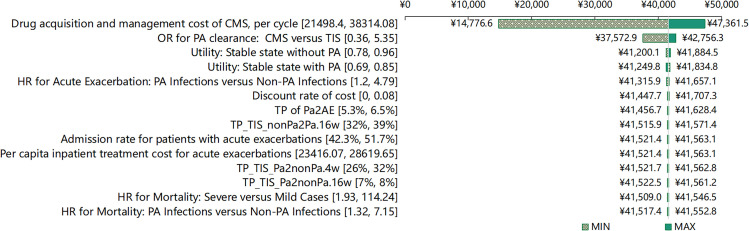
Tornado diagram showing the results of one-way sensitivity analyses for the base case in China’s healthcare system perspective.

Fig 2 presents the ten most influential sensitivity analyses comparing TIS and nebulized CMS. Throughout all scenarios, the net benefit of TIS ranged from CNY 14,776.60 (USD 2,044.98) to CNY 47,361.50 (USD 6,554.50). The ICER was particularly sensitive to variations in the Drug acquisition and management cost of CMS, the OR of PA clearance for CMS versus TIS, and Utility of Stable state without PA infections. These findings consistently affirmed TIS’s economic superiority over nebulized CMS across the current range of parameter variations.

### Probabilistic sensitivity analysis

**Fig 3:**
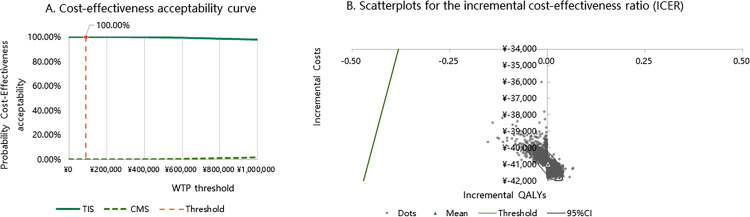
Probabilistic Sensitivity Analysis. (A) Cost-effectiveness acceptability curve. (B) Scatterplots for the incremental cost-effectiveness ratio (ICER) from the perspective of China’s healthcare system for the base case.

The PSA exposed cost-effectiveness acceptability curves and ICER scatter plots, illustrated in Fig 3. At the WTP threshold of CNY 89,358 (USD 12,366) per QALY, the probability of TIS being cost-effective reached 100%, whereas it remained at 0% for CMS nebulization, underscoring the robustness of the base-case analysis.

### Scenario analyses

Scenario analyses explored variations in the duration of the length of the time horizon. Simulations over varying time lengths demonstrated that, from the perspective of the healthcare system and at a threshold of one times the national per capita GDP, TIS consistently proved to be less costly and more effective than nebulized CMS, as shown in S3 Table.

## Discussion

In China, despite the low prevalence of BE compared to other respiratory diseases like Chronic Obstructive Pulmonary Disease (COPD) and asthma, the large population base contributes to a significant healthcare burden [1]. Inhaled antimicrobial medications, which achieve high local concentrations in the lungs while maintaining low systemic exposure, offer both efficacy and safety benefits for BE patients. Only two Inhaled antibiotics are available in China, so it is significant to evaluate the cost-effectiveness between two drugs.

According to previous published studies, the therapeutic outcomes of inhaled tobramycin are consistently superior to those of inhaled Colistin as a prolonged inhalation treatment in patients with PA-infection BE, which was evidenced by a decrease in PA density, an increase in PA clearance rate, a reduction in the frequency of acute exacerbations, and the improvement of lung function [10,16,17,21]. In this study, TIS, compared with nebulized CMS for Chinese patients with BE and PA infections, proved to be an economically dominant strategy. It showed a net benefit ranged from CNY 14,776.60 (USD 2,044.98) to CNY 47,361.50 (USD 6,554.50), with a 100% probability of being cost-effective at the WTP threshold of CNY 85,698 (USD 11,860) per QALY. Obviously, TIS represents the optimal choice for the management of PA clearance during the stable phase in BE patients with PA infection, both in terms of efficacy and cost-effectiveness.

Despite guideline recommendations favoring both TIS and nebulized CMS as key treatments for BE, there has been a notable lack of relevant economic evaluations. Consequently, a direct comparison with cost-effectiveness models for similar therapies has not been feasible. For instance, *Bhattacharyya S.B.* [27] developed a four-week Markov model from a UK healthcare perspective to predict the lifetime clinical and economic outcomes for BE in adults. This model, featuring one stable disease state and three acute exacerbation states, demonstrated that treating BE could reduce medical costs by 10,777 pounds and yield an additional 0.11 QALYs. Similarly, *Christin* [28] developed a decision tree/Markov model estimating the cost-effectiveness of tiotropium bromide versus placebo over one year for adult patients with BE and airflow obstruction. The findings indicated a gain of 0.03 QALYs and a reduction of 0.01 exacerbation events with tiotropium, at an incremental cost of 137 New Zealand dollars, leading to a cost of 12,896 New Zealand dollars per exacerbation avoided and 4,655 New Zealand dollars per QALY gained.

While these previous studies provide valuable insights into BE treatment, several limitations warrant careful consideration. For example, *Bhattacharyya S.B.‘s* study, while offering a comprehensive lifetime cost and outcome simulation, employed a simplified model that may not fully capture the complexity of long-term health conditions and disease progression. Additionally, *Christin’s* research, though insightful for patients with airflow obstruction, relied primarily on short-term data, which may not accurately reflect the cost-effectiveness of long-term treatment strategies.

The current economic evaluation benefits from the utilization of updated, patient-level clinical trial data to model transitions between stable PA states, thereby minimizing unnecessary assumptions. Moreover, the utility values for stable PA and acute exacerbation states were derived from EQ-5D-5L scores reported in post-marketing clinical studies comparing TIS with nebulized CMS, providing a more accurate reflection of the real preferences of patients in clinical trials.

However, this study has its limitations. Due to the absence of disease utility studies for BE patients in China, utility values for stable states without PA were extrapolated using formulas from Korean population, potentially introducing bias.

## Conclusion

Our comprehensive analysis robustly demonstrates the cost-effectiveness of TIS compared to standard nebulized therapies for managing BE with PA infections in China, highlighting its potential to mitigate both the economic and clinical burdens of this chronic condition. These findings not only support the inclusion of TIS in clinical guidelines but also underscore the critical need for continued research and policy development to enhance patient care in BE. Future research should extend the treatment duration and include larger patient cohorts to solidify our conclusions and guide the efficient use of healthcare resources more effectively.

## Supporting information

Below is the link to the electronic supplementary material (Supplementary file).
